# Understanding parental perspectives on young children’s oral health (≤ 4 years) growing up in a disadvantaged neighbourhood of Amsterdam, the Netherlands: an exploratory study

**DOI:** 10.1186/s12889-024-18073-0

**Published:** 2024-02-27

**Authors:** Awani Balasooriyan, Monique H. van der Veen, Clarissa Calil Bonifácio, Christine Dedding

**Affiliations:** 1 Department of Preventive Dentistry, Academic Centre for Dentistry Amsterdam (ACTA), University of Amsterdam and VU Amsterdam, Amsterdam, The Netherlands; 2 Department of Paediatric Dentistry, Academic Centre for Dentistry Amsterdam (ACTA), University of Amsterdam and VU Amsterdam, Amsterdam, The Netherlands; 3 Department of Ethics, Law and Humanities, Amsterdam University Medical Centre (UMC), Amsterdam, The Netherlands

**Keywords:** Children, Parents, Child oral health, Family life, Vulnerable circumstances, Oral health inequality

## Abstract

**Background:**

Families’ understanding towards oral health problems among young children is poorly studied. More insight into parents’ experiences, especially of those living in disadvantaged neighbourhoods, is needed to address persistent oral health inequalities. This qualitative study aims to explore parental perspectives on children’s oral health (≤ 4 years) and the opportunities they see to improve children’s oral health.

**Methods:**

Forty-seven mothers and five fathers with different migration backgrounds from a disadvantaged neighbourhood in Amsterdam, the Netherlands, participated in our study. Semi-structured interviews (*n* = 27), participant observations (*n* = 7) and one focus group discussion were conducted. A thematic data analysis was used.

**Results:**

Parents describe their daily life with young children as busy, hectic and unpredictable. Parents seem to be most concerned about parenting. Mothers, in particular, feel fully responsible for raising their children and managing daily complexities. While most parents value their children’s oral health, they all experience challenges. Parents find it hard to limit daily candy intake and to handle unwilling children during tooth brushing. They feel limited support for these issues from their household, social network and professionals.

**Conclusion:**

Parental struggles in children’s oral health are complex and interrelated as they occur across family, societal, community and professional levels. Given the complex daily reality of families with young children, establishing and maintaining healthy oral health habits seems not at the top of parents’ minds. They ask for advice in the upbringing of their children backed up by social support, increased attention to children’s oral health within the community and professional assistance. Collaborating with parents as knowledgeable partners might be the first step in acting upon the endeavour to address oral health inequality among young children.

**Supplementary Information:**

The online version contains supplementary material available at 10.1186/s12889-024-18073-0.

## Background

The high prevalence of oral diseases during childhood poses a serious global health threat [1]. Despite being preventable, dental caries is the most common oral disease affecting millions of young children worldwide [2]. Children with caries in their primary teeth are more prone to developing oral diseases and other health problems later in life [3]. In addition to the health effects, caries may burden a young child’s quality of life, leading to high costs for families and society [4]. Moreover, caries frequently affect groups living in vulnerable circumstances, such as children growing up in disadvantaged neighbourhoods [5, 6]. Childhood caries mirrors socioeconomic and oral health inequalities [1, 7]. Therefore, promoting and adopting healthy oral health behaviours early in life is urgent.

In their recent Global Oral Health Action Plan, the WHO defined oral health inequalities as “*differences in oral health status that are avoidable and deemed to be unfair, unacceptable and unjust*” [1]. In a study conducted in four cities in the Netherlands, Schuller et al. (2019) showed that caries levels among 5-year-old children from low socioeconomic position (SEP) families were 29% compared to 19% in the high SEP group. Large oral health inequalities are reported within the low SEP group: 74% of the 5-year-olds with a maternal migration background experienced caries. In comparison, caries prevalence was 22% when their mothers were born in the Netherlands [8]. Although dental costs for children in the Netherlands are reimbursed up to the age of 18, access to dental care is limited [9]. In 2021, 56.6% of children under the age of four years, whose socioeconomic position or migration background is unknown, did not visit the dentist.

Research has increasingly focused on better understanding the broader social determinants causing oral health inequalities [10]. This includes political context, access to health care, social support, housing conditions, socioeconomic position and parental factors [11–14]. For instance, Baker et al. (2018) found that politics, social and public policy influence children’s oral health [15]. Van der Tas and colleagues (2017) demonstrated higher caries levels in 6-year-old children of parents with a low educational level, low household income, and unemployed [16]. These insights contributed to the design of population-level interventions, often implemented by a top-down approach to reach equity in oral health across a population [17–19].

Although researchers expected that these population-level interventions would minimise oral health inequalities, several studies show differently [20, 21]. Levin et al. (2009) found that a school-based fluoride mouth-rinsing program led to significantly lower caries levels among children from the least deprived backgrounds [22]. Tubert-Jeannin and others (2012) demonstrated that an Oral Health Promotion program had minimal effect in reducing children’s oral health disparities [23]. In addition, according to Qadri and co-workers (2019), an oral and general health promotion intervention significantly impacted children from high SEP families [24]. Scholars argue that population-level interventions may unintendedly have widened young children’s oral health inequality gap [12, 18]. Therefore, combatting persistent oral health inequalities appeals to a different approach.

To design an effective child oral health promotion intervention, it is imperative to collaborate with families and prioritise their perspectives [25, 26]. We need to step away from the conventional ‘top-down’ approach and acknowledge the importance of a ‘bottom-up’ approach during intervention development and implementation [27]. This demands insight into families’ lived experience towards oral health problems as they hold vital knowledge of their daily circumstances, needs and contextual interactions [28, 29]. Unfortunately, this knowledge is still scarce, and a deeper understanding of a family’s complex daily reality is needed [30–32]. Families living in vulnerable circumstances are seldom approached as knowledgeable stakeholders, and their voices are often unheard during intervention design processes [33, 34].

This study aims to explore parents’ perspectives on oral health of young children (≤ 4 years) growing up in a disadvantaged neighbourhood in Amsterdam, the Netherlands, and to solicit their advice on how to improve their children’s oral health. The results will be used to educate professionals within and outside the dental sector, to co-develop an oral health promotion intervention tailored to families’ needs and daily context, and to identify future research lines.

## Methodology

### Study design

This qualitative study is the first step in a larger Participatory Action Research (PAR) project. This PAR project aspires to collectively learn with all stakeholders – parents and caregivers, professionals within and outside the dental sector, and policymakers – and to co-create effective interventions to improve oral health of young children (≤ 4 years) living in disadvantaged neighbourhoods in Amsterdam, the Netherlands. The perspectives of professionals towards young children’s oral health are explored in another study [35]. In this study, we focused on the perspectives of parents with young children (≤ 4 years) by using qualitative research methods that are underpinned by the conceptual framework of Fisher-Owens and others (2007) [11]. This multilevel conceptual framework includes five domains of determinants on child-, family- and community levels that may influence children’s oral health outcomes. The framework formed the foundation for designing the interview guide and tailored work plan to understand better parental views on children’s oral health and the complex interaction between the risk factors at multiple system levels. This study is conducted in Amsterdam New-West, a neighbourhood with a relatively high number of households with a low SEP, a migration background and problems with housing, health, and employment [36, 37]. In 2018, 71% of the children aged four years or younger did not visit the dentist in this neighbourhood [38].

### Participants

We focused on parents currently raising young children (≤ 4 years) and living in Amsterdam New-West. We aimed to include a diverse group of parents in terms of family composition, household functioning, occupation, cultural background and social network. Parents, who bring their children to a preschool or playgroup in Amsterdam New-West, were also considered eligible.

To meet parents, the first author (AB) participated in various free community activities organised by local societal organisations. AB had breakfast with a group of Moroccan women, explained the study’s aims and what participation entailed. Also, AB joined several playgroup sessions, informed parents about the study’s goal and answered parents’ questions about their children’s oral health. AB was mindful of the cultural significance of what parents said and made valuable reflexive notes to gain a sufficient preunderstanding of the families and their context. With the help of local community professionals and the personal network of our research team, we invited parents to participate in our study. Besides, we spoke with parents in the waiting room of a local paediatric dental practice while their child was being treated under deep sedation. Data were collected until data saturation was achieved [39].

### Data collection

In total, 47 mothers and five fathers participated in our study. Data were collected between March 2022 and March 2023 through (group) interviews, participatory observation and one focus group. All qualitative methods were led by the first author (AB) and assisted by students with a dental health background (NR, AN, AJ, MT, MG) or a general health background (SD). AB is experienced in qualitative research through training and previous research projects. Data were collected in Dutch (*n* = 45) and English (*n* = 3) or with an interpreter who translated from Arabic to Dutch (*n* = 2) or Turkish to Dutch (*n* = 2). A summary of the main findings was returned to the parents for member checking. A description of each qualitative method is given below.

1. Interviews. We conducted 24 individual interviews, two group interviews with three participants and one duo interview with a father and a mother. Interviews were held in a home setting, paediatric dental practice, preschool, local community organisations, playgroups, mosque, dental school or via telephone. A semi-structured interview guide was developed based on initial conversations with parents and previous research [35]. The interview guide was critically reviewed by all authors and pilot-tested with the research team. We asked parents about their daily lives, perspectives on oral health, experiences with poor oral health in young children, their needs and possible solutions. The interviews lasted between 30 and 70 min.
2. Participant observations (*n* = 7) lasting between 30 and 60 min. We joined parents and children during playgroups and a communal breakfast for mothers at a local community centre. The number of participants ranged from three to fourteen mothers. We used pictures of a young child with tooth decay and examples of (un)healthy food to talk to parents about different aspects of children’s oral health. We observed how they talked about their daily family life, oral health and associated problems, parenting strategies and informally discussed opportunities to improve oral health in the neighbourhood.
3. Focus group discussion (*n* = 1). We collaborated with one preschool to discuss child oral health topics in which five mothers participated, lasting one hour. We followed a tailored work plan, consisting of pictures of a young child with cavities and examples of (un)healthy food, to gain insight into family’s daily living circumstances, dietary practices, perceptions of child’s oral health, difficulties in establishing good oral health habits and on how this can be improved.

### Data analysis

Data from 20 (group) interviews (*n* = 25), six participant observations (*n* = 12) and one focus group discussion (*n* = 5) were audio-recorded and transcribed. Extensive notes were made in seven interviews (*n* = 7) and one participant observation (*n* = 3). First, the data were inductively analysed by using the coding software ATLAS.ti. Windows (version 9.0.19.0, Scientific Software Development GmbH in Berlin, Germany) to develop an initial coding scheme. We followed the thematic analysis principles of Braun and Clarke (2006): (1) familiarisation; (2) initial coding; (3) searching for themes; (4) critically reviewing themes; (5) defining themes, and (6) producing the article [40]. By (re-)reading transcripts and notes, we reviewed the initial coding scheme and adjusted to new insights. Overlapping codes were organised into broader themes while staying aware of the interrelatedness and complexity of the themes. The final themes were critically discussed with all authors (MV, CB and CD) to evaluate whether the data supported the key findings, interpretations and conclusions. To avoid any loss of meaning in the Dutch quotes, the quotes were translated into English during the final phase of preparing the manuscript.

## Results

The group of participants is diverse in terms of their role (47 mothers vs. five fathers), cultural background (Brazilian, Dutch, Eritrean, Moroccan, Philippian, Syrian, Turkish or unknown), employment status (full-time, part-time, or unemployed), number of children per household (1 child up to 7 children) and relationship status (single, married or unknown). Participants were invited via local preschool (*n* = 14), playgroup (*n* = 14), societal organisations (*n* = 6), personal network (*n* = 9), paediatric dental practice (*n* = 4), mosque (*n* = 3) and library (*n* = 2). Additional File 1 shows a complete overview of the participants.

From the analysis of the data, four themes were derived. Parental views on (1) Daily lives of the families; (2) Meaning of young children’s oral health and associated problems; (3) Parents’ struggles in young children’s oral health; and (4) Opportunities and strategies for child oral health promotion.

### Family life with young children is hectic

Most parents say that parenting is not easy. A 27-year-old mother, currently unemployed and taking care of two young children (three and one-year-old), describes parenthood as “*It’s the most beautiful thing there is. But it’s also the toughest thing there is […]. Yes, I am very happy. I think it is a very nice situation we are in now, but it is sometimes tough […]. Parenting is just a profession in itself*.” A Moroccan mother expresses her concerns about child rearing: “*I have [3 children] now, and I am just grateful, but it is very difficult for parenting. […] Parenting is very difficult at this time.*” A single mother with a 3.5-year-old son adds: “*Being a mother is heavy. Parenting is not easy*.”

In most families, fathers work more than mothers do. Therefore, mothers feel highly responsible for their children’s upbringing. A mother with two children describes how she is mainly involved in the upbringing of her two children because of her husband’s working hours: “*I actually do most of it by myself, so at some point, it’s just in there, so I know what I’m doing because he is not around as much.*” Most mothers do not want to burden their husbands because they have “*little energy*” at the end of the day. A Turkish mother says that her husband sometimes helps with parenting, but she adds, “*He does not spend 24 hours with him, he works, and when he comes home, he also says, ‘I’m tired too’*.” A working mother with three children does not expect help because: “*I already assume that I just have to do it on my own. And if that involves help from my husband, then that’s fine. But I’m not necessarily waiting for it*.”

Mothers share their difficulties in managing their busy and hectic daily lives. They describe an ordinary day as “*intensive*”, “*a very long day*”, “*a full program*”, “*too little time”*, and “*exhaustive*”. Mothers run around getting their children ready for the day, bringing them on time to preschool or school, picking them up at specific times and taking them to other activities. A mother from Moroccan origin struggles to combine the upbringing of her two children with working. She says, “*It’s always a lot of scheduling and arranging*.” Multiple mothers notice that children are nowadays very demanding. A married mother wants to provide enough attention to her youngest daughter but struggles with being fully responsible for the upbringing of her three children and doing all house chores. Mothers do their best to keep structure in their family. An employed and married mother with three children (four, two and one-year-old) tries very hard to maintain structure but:

*“You don’t know what can happen. You have broken nights, you have weepy kids in the morning, accidents that can happen, exploded nappies, and all those things affect your schedule, causing you to get completely bogged down with your daily schedule in the morning.”*

Parents also say that living in an area characterised as a disadvantaged neighbourhood influences their family life. Mothers complain about the poor housing facilities and current building renovations in the neighbourhood. A pregnant and unemployed mother talks about all the mould in her small student apartment: “*Whole bathroom wall, everywhere full of mould*”, which she shares with her son (nine months) and husband. A Moroccan mother frustratedly says: “*And walls are all broken, a lot of dust, and that is actually not healthy for me and also for my children. My youngest child got eczema from a lot of dust*.” On the contrary, a married father is happy about his 60 m2 old apartment, which he shares with his three children, and he adds, “*Amsterdam with a single-family home is a bit difficult you understand, so unfortunately, getting single-family home is not for everyone*.”

### Child oral health is important, but…

Most parents value their children’s oral health when the topic is raised. For parents, oral health in young children means “*healthy teeth*”, “*good brushing*”, “*healthy food and drinks”*, and “*not too many sugars*”. A mother born in the Philippines finds oral health of her 19-month-old daughter important because “*good teeth make you smile*”. A Syrian mother with five children emphasises in Arabic a good oral health of her youngest son so that “*He can eat enough, [he] can always enjoy his smile, if he is not in pain, if his mouth is not dirty […] then he can have fun with everything*.” A mother highly values her daughter’s oral health because of her own problems: “*I think it is important to brush consciously, so I do all the oral health care. So that she doesn’t get such problems with her teeth, I think it’s extra important for her.”* A father adds:

“*From childhood, oral health is actually the most important thing there is. So we make sure that all of us, so children as well, brush the teeth properly, […] it’s a bit of getting the child used to it from an early age that later on the child will grow up in such a way that they don’t actually say later on of ‘oh yes today brushing teeth, no don’t have to’*.”

However, a few parents question the importance of children’s oral health. A Turkish mother notes: “*Yes, it is important […] I don’t want him to have cavities later of course*. *But I’m like, it’s more important after the baby teeth. Those teeth are going to come out anyway.*” In a discussion about the relationship between nutrition and teeth, a pregnant mother with seven children says: “*Teeth is not in the first place actually, does come second, first place is surely what it does to your body, second place is teeth.*” Another mother finds oral health important, but simultaneously mentions that her son’s oral health has a lower priority than playing and his evening meal.

Although parents differ in the perceived importance of their child’s oral health, they all experience oral health problems within their household. A mother expresses her disbelief towards the cavities of her daughter: “*[I] brushed many times, and really twice a day, she only drinks water, and still she still has these cavities, and I also just helped there with brushing, it could also just be something, yes, maybe genetic, and even though you do your best*.” Another mother frustratedly says that her son experienced cavities at a young age while he didn’t eat candy. A mother talks about oral health problems when her daughter was four months old: “*She had gum inflammation. And I didn’t know how that came because it was an awful infection. She was in a lot of pain.*” A Moroccan father describes his three-year-old son’s poor oral health while his son is being treated under deep sedation in a paediatric dental practice: “*[He] has four big holes, eight molars in the back, then next to that he also has holes, and then the front teeth, he also has all these holes […] So, or in other words, his whole set of teeth is 0.0*.”

### Parental struggles related to child oral health

#### Limiting candy is a daily challenge

Parents struggle with their children’s preference for candy or unhealthy food. A single mother mentions about her son: “*He is really picky with food. He prefers just to eat candy all day*”, and she adds that candy is given out of love. Young children tend to ask for candy continuously throughout the day. A mother frequently gives candy to keep her daughter calm so that she can finish her daily house chores. A father with a Moroccan background and five children expresses his concerns: “*I always struggle with those fruits and vegetables, struggling for those kids*.” And a mother adds: “*My children, I can’t control them when there is candy at home*.”

Sometimes, controlling children’s candy intake is complicated due to parental clashes. A mother explains: “*I don’t buy candy, but my husband also has a bag of candy on top of the cupboard, so once in a while, he gives all the kids candy, too.*” Mothers feel that their husbands are more easygoing when offering candy to their children. A Turkish mother frustratedly says her husband tends to give crisps to her children in the morning before breakfast. Another mother, whose three-year-old son has eight cavities, finds it difficult to disrupt her son’s “*daddy-moment*”, in which they eat unhealthy food just before sleeping.

Also, parents feel challenged by the presence of unhealthy food during family visits. A mother from Moroccan origin describes how she has to control her “*sweet-toothed*” daughter when visiting grandparents: “*Then there is cake on the table, then there are biscuits on the table […] if you don’t pay attention for a moment, she has already finished three of them*.” Parents struggle to deal with the need from family members to provide young children with something sweet. A mother mentions: “*We were at a party […] at my husband’s family, […] then he [my youngest] got a whole bounty or a snickers in his hands, I almost had a heart attack*.” Confronting family members in giving candy is perceived to be hard, as a mother explains:

“*I also don’t know what to do when someone gives him something. ‘Yes, give it back’. I just find it awkward. While I see plenty of parents who do so. I feel ashamed again. I find it awkward again to do that in front of someone*.”

Some parents perceive Amsterdam New-West, as an unhealthy living environment. A mother struggles to find “*easy-accessible healthy food*” when having a day out with her three-year-old son. She experiences differences in the availability of healthy food between different neighbourhoods in Amsterdam. A mother who recently moved to Amsterdam New-West recognises this: “*I have neighbours here who very often bring candy, which is very sweet for my children […] you just really notice that difference between New West and South […]. Once, I got a book for my son there*.” On the contrary, a father believes that living in an unhealthy environment does not influence cavities among young children because “*Everywhere you go in the Netherlands you have snack bars, whether it’s in New West or Old South […] it all depends on yourself […] It’s not the snack bar’s fault*.” A Surinam-Dutch mother agrees, saying, “*Look, of course, there are many snack bars, but you don’t do your groceries there*.”

#### Difficulties with toothbrushing

Many parents express their frustrations with tooth brushing in young children. Children tend to “*yell*”, *“cry”, “vomit”, “scream,”* or “*refuse*” during tooth brushing. A mother talking about her son: “*He says: ‘mummy, I’m getting nauseous, no, stop’. Then I have to stop right away*.” Young children often do not want to be helped by their parents, resulting in a fight. A mother illustrates: “*If I want to brush his back molars, he puts his jaws together, and yes, you try to brush, then you sit there all the time fighting, crying, you name it*.” A Dutch mother with a four-year-old daughter adds: “*It’s really not fun, you know, […] and then you don’t want that daily hassle around teeth brushing, you know, that you think ‘get those teeth apart now’*.”

Sometimes, parents feel too tired to handle their children’s behaviour. A mother tries her best to help her youngest children with brushing, she explains, “*Then you get a struggle, and then I get tired, and then I think ‘yeah, bye, just take it easy, you know, never mind’*.” A mother adds that her son does not feel like brushing his teeth in the evening because “*He’s just tired, then I’m tired, so then I think ‘never mind’*.” Another mother mentions that she does not help her three children during brushing when she feels too tired. A mother with a Turkish background feels guilty about feeling tired and says, “*It’s only a one-minute work, ‘Couldn’t I keep it up a minute longer?’ Afterwards, you get that feeling of guilt, ‘What if they are going to get cavities?’*.”

Due to limited time and busy schedules, parents tend to skip tooth brushing. A mother of three children explains: “*I try twice, but it doesn’t always happen in the morning, I must say, because of the rush and bustle. And I must say that I regret that*.” According to a father, “*rush hour in the morning*” is the biggest problem. He adds, *“Then they have to eat, and then they have to get dressed et cetera, if we then think ‘we don’t have much time’, then not today.”* Brushing is also skipped after a “*tough day*” or a “*long hard day*” and illustrated in Turkish by a mother:

*“You run there and then there again, and you are completely exhausted at the end of the day. A person gets very tired from that, so even for brushing your teeth, there is no time left, so then you would just prefer to lie in bed*.”

#### Limited support in children’s oral health

Often, mothers pay the most attention to establishing good oral health habits for their children and lack support from their husbands. A Moroccan mother explains, *“My husband really doesn’t think about that [her daughter’s teeth] […] he thinks ‘whatever’ […] Yes, in things like that, I’m the one that says, ‘hey, we should make an appointment at the dentist’*.” Another mother talking about her husband: “*It’s not 123 that it occurs to him of ‘I’m going to take my child to the shower, and I’m going to brush his teeth’*.” Multiple mothers describe their important role in their children’s oral health: “*If the mother doesn’t think about it then it doesn’t happen […] It doesn’t even cross the father’s mind*.” A mother with three daughters mentions that her husband is supportive in doing house chores, but his help in oral health is lacking.

In contrast to topics like breastfeeding, playing or screentime, child oral health is rarely discussed with friends and family. A mother argues that oral health might be a “*boring topic*” and elaborates, “*I don’t know why we don’t talk about it unless someone has a pain, a toothache*.” A father finds discussing his children’s teeth uncommon during daily conversations because “*If his tummy is bothering him, you do start talking about it […] you don’t start talking about, ‘how often do you brush your child’s teeth?’”* A mother believes tooth brushing “*is something normal*” and “*nothing new*” to discuss. Another mother recognises this, arguing that everybody automatically assumes everyone brushes his or her own teeth. It might even be a “*taboo*” to talk about bad teeth. On the contrary, a Dutch mother heard from several parents that they hate brushing their children’s teeth.

Parents see limited support from general dental practices as a barrier to taking their children to the dentist. Not all dentists advise that brushing and dental visits from the first tooth are possible. A mother did not know she could bring her young children to the dentist and adds, “*I learned something again. Actually, I can bring my baby along too*.” Differences in dental policies make it unclear to parents when to register their children. A mother struggling to brush her two-year-old daughter regrets not being informed earlier because her dentist said to bring a child “*From one year, so they get used to the environment*”. While another mother received the advice: “*Dentist really said to me only from four years old*.” Sometimes dentists are reluctant to treat young children because they may not cooperate. A father is unhappy about general dentists because “*They only look at the money and not the teeth*.” An Italian mother adds: “*I was actually surprised how little the dentist knew about the young children’s paediatric dentistry, so that was shocking*.”

Finally, parents experience too little help from professionals working in general health care, child health clinic, child daycare and preschool or school. A father from Moroccan origin expresses his frustration towards school by saying, “*School never talked about the fact of tooth brushing […], but they do talk about nutrition*.” Not all parents might know about the potential influence of nutrition, including prolonged breastfeeding (> 12 months) and bottle feeding during the night, on their children’s teeth. A mother who works as a dental hygienist says, “*I didn’t know that breastfeeding causes a lot of cavities. I really didn’t know, with my first one, he had so many cavities because he didn’t want to brush properly, but he was also breastfed until two years*.” Besides, parents share different stories about the child health clinic. One mother is puzzled because “*the child health clinic, they never informed us about dentist or anything either, we really had to figure that all out on our own*.” In contrast, other parents indicate that the child health clinic advised them to start brushing from the eruption of the first tooth.

#### Opportunities and strategies to improve oral health

Parents propose different strategies that could help them to handle their unwilling children and limited support from their surroundings, some of these strategies they already use, others they need to remind themselves of or even wish to receive help with. Parents see opportunities in parenting, family roles, social support, community approach and professional assistance.

#### Parenting

According to parents, being consequent and setting clear rules is important to limit children’s daily candy intake. A Turkish father argues: “*As a parent, you are responsible. You should not buy [candy] or buy less of it.*” Another father adds: “*If you say ‘yes’ today, then the next day they are going to ask the same […] again, ‘oh, if I do that, then maybe I can get some extra candy […], so you have to stick to the limit yourself*.” Parents find explaining to their children how candy may result in cavities or pain is useful. A mother says that her two children ask less for candy because they say, “*Sorry mum […] No, my teeth will hurt […] I’m not going to say any more ‘I want more [candy]’*.” A mother acknowledges that she needs to be more strict regarding candy but wishes for other alternatives for her child. A father would like more help with what candy does, and he questions: “*What’s bad about candy? How much should you take per day? What kind of candy is good?*”

To address tooth brushing difficulties, parents agree that perseverance is key. Parents advise to include brushing step-by-step in a daily routine from early on by making it more “*fun*” and *“playful*” for young children. Brushing together with siblings and making it part of the sleeping ritual helps to deal with the daily hectic family life. A mother describes how her five children enjoy brushing together. Besides, parents use books, sandglasses, music, toys and songs to make brushing more fun. For instance, a mother buys “*toothpaste with nice things on it*.” Another mother stresses the importance of explaining to her three children what she does during brushing. A father believes forcing his son is useless, but you should “*Stand behind [the child], helping, supporting, listening.*” A mother with a Moroccan background strives to *“just do my best then […] in terms of brushing my teeth, just no excuses, get up and just do it*.” Most parents would like to receive practical tips on how to deal with unwilling children during brushing.

#### Family roles

Parents mention that it is important to engage with each other to limit their children’s candy intake. Parents propose different strategies to address their discordance in offering candy. Mothers confront their husbands or ensure that candy is not visible to their children. A Moroccan mother mentions: “*Dad really loves his candy too. And I always try to hide his bags in a very tall basket in my cupboard*.” A father says he and his wife agreed to be strict towards their 1.5-year-old son. Another father admits that he should consider his own behaviour: “*Again, no more candy, so that means I can’t buy candy either*.” Mothers especially wish for more support from their husbands by sticking to the agreements on food. One father is eager to stop his three-year-old son’s candy intake by buying it once a week but needs help because “*You can’t forbid them. That’s impossible*.”

Parents believe both father and mother can help deal with unwilling children during tooth brushing. There are households where parents are equally involved in tooth brushing. A mother describes her husband’s role: “*When we have a brushing moment, he takes one child, and I take one child, so then we brush together on the sofa*.” Fathers also help upon request, and a Moroccan father explains how he encourages his five children: “*You have to listen, your mother said ‘go brush your teeth!’ I’m just his mother’s helper, you know*.” Some mothers feel that their husbands cannot brush properly. During a community breakfast, a mother mentions that her husband is not allowed to brush her five sons’ teeth because “*His brushing is another way of brushing*.” A majority of the mothers would be happy to receive more support from their husbands without arguing or bothering them.

#### Social support

Parents say that having conversations with friends and family is useful in limiting candy. A mother talks about how she confronts her sister to stop giving candy to her son: “*You should stop with candy […] there should just be no option.*” Sometimes, children are allowed to accept candy from family members, but parents ensure they don’t open it or immediately take it away. Although parents try to engage with grandparents to address their indulgent behaviour, they find it difficult. A Turkish mother struggles to convince her father to consider his behaviour because “*I can also hardly go yelling at my father*.” Another mother aims to motivate herself and her friends to put grapes and other fruits on the table when they drink tea with the children. Most parents want help in dealing with the embedded need of their social surroundings to provide something sweet to young children.

#### Community approach

Parents perceive “*social media*”, “*advertisements*”, and “*handing out free oral health materials*” as valuable to increase awareness of children’s oral health within their community and neighbourhood. Parents learn from each other when they discuss oral health topics at activities organised by local societal organisations. During a communal breakfast with fourteen women, a mother demonstrates how she ensures that her children’s teeth are brushed. Everyone perceives her practical oral health tips as useful. The mothers of the playgroup find it helpful to hear how one mother describes her strategy to brush with her three children collectively: “*I sit on the floor and then I do them one by one […] then they lie down, and then I start brushing, and then the next one comes, the next one and so it goes*.” Including oral health in existing neighbourhood events is promising to improve attention within the community. For instance, a Moroccan mother talks about handing out toothbrushes and toothpaste at a well-attended neighbourhood fair by families. Parents generally appreciate help through a community approach to establishing healthy oral health habits.

#### More professional assistance

Parents see opportunities in the current procedures of general dentists. They believe listening to families’ needs and giving advice without judgment contributes to access to dental care. In addition, parents prefer a dental appointment every three months, as illustrated by a mother: “*Then I’ve been to the dentist, and it goes well again for a month or two, and then I actually lapse back into the same behaviour again*.” Most parents praise the presence of a school dentist. A Moroccan father expresses his preference for a paediatric dentist by saying, “*I don’t want to take [them] to a normal dentist either, but rather to the paediatric dentist, because they know better how to deal with children than normal dentists do*.” Parents reason that general dentists should be more “*friendly*” and “*patience*”, and show “*empathy*” when treating young children. Most parents need general dentists’ assistance handling their unwilling children during brushing. A mother who regrets that she was not informed to bring her daughter to the dentist mentions: “*I think it’s a pity even though I […] make the efforts to brush my daughter, I have to have a professional to guide me a little bit in that*.”

Furthermore, parents believe that maternity care, general practitioners, child health clinics, child day care, playgroups, preschools and schools, and the municipality should pay more attention to child oral health. Multiple parents propose ideas for preschools, such as “*collective tooth brushing in the morning*” or introducing “*oral health playfully as a theme*” to children. Parents also appreciate the existing food policies of the school as explained by a mother: “*Nowadays you are only allowed to bring water or milk […] Biscuits in the afternoon, and things like that are all no longer allowed, so that helps already*.” A mother believes the child health clinic could briefly discuss brushing and limited candy. A mother with a Moroccan background agrees: “*I think it should actually be included in the check, in the body check of a child, because the eyes are also checked, the ears are checked, and only the teeth are missing.*” Parents would like to receive help from professionals outside the dental sector to understand, for instance, when breastfeeding becomes unhealthy for their children’s teeth and other nutritional influences on oral and general health.

Finally, parents say that collaboration between different professionals can be promising. An example is to hire a dentist at preschools and schools to reach families and inform them of the importance of child oral health. A mother explains this: *“It would just be nice if they [preschool] also talked about that, that a dentist really comes by once you know, that kids really grow up with it, that it becomes visible*.” A Moroccan father argues, “*Parents, general practitioner, school and dentist, they need to work together*.” And a mother adds:

*“So maybe those agencies, the childcare, preschool, if we want to tackle it early on, that we should also seek a bit of cooperation there, that the dentists will do a bit in that way, and of course also the education from the child health clinic*.”

## Discussion

This exploratory study is one of the first doing right to the everyday complexities of families from a disadvantaged neighbourhood, how this might influence parents’ perspectives on their children’s oral health (≤ 4 years), and the opportunities they see to improve their children’s oral health.

Our results show that oral health seems not at the top of parents’ minds, mainly because their daily lives with young children are busy, hectic, and unpredictable. Parents seem to be more concerned about parenting. Mothers, in particular, feel fully responsible for raising their children and managing daily complexities. While most parents value their children’s oral health, they all experience challenges. Parents find it hard to limit daily candy intake and to handle unwilling children during tooth brushing. They feel limited support regarding these issues from their household, social network and professionals. Parents ask for help in raising their children, backed up by social support, increased attention to children’s oral health in the community and professional assistance.

First, we found that oral health may not be a priority in the hectic lives of families with young children. A few qualitative studies support our finding that oral health may be unintentionally given a lower priority due to everyday complexities [41–43]. Isong and co-workers suggested that competing priorities had pushed children’s oral health down the list of other tasks [41]. Similarly, Nayee and colleagues (2018) identified parents from a deprived area in London as ‘Oral Health Non-prioritisers’ based on the perceived irrelevance of oral health to overall health and irregular dental visits [43]. The parents in our study seem to be more concerned about parenting. However, managing daily complexities is a major challenge for good parenting. It is well-known that parenting styles strongly influence children’s oral health [11, 44]. Duijster and co-workers (2015) state that parental confidence in controlling their child’s oral health and positive parenting practices lead to lower caries levels [45]. Helping parents to establish healthy oral health habits for their children demands an approach considering the complexities of parenting and everyday life.

Second, most parents value their children’s oral health but struggle to limit daily candy intake, handle unwilling children during tooth brushing and feel limited support regarding these issues. We, and other scholars, have emphasised that parental struggles are complex and interrelated as they occur across family, societal, community and professional levels [11, 25]. The indulgent behaviour of the social environment in providing candy to young children is found in several studies [42, 46]. According to Burgette and co-workers (2023), mothers weigh the benefits of protecting their children’s oral health against the costs of potential conflict when confronting grandparents [46]. Limited support by dentists and other professionals is also described in the literature [31, 42, 47]. Mothers participating in the study of van Nes and others (2018) complained about the dental working procedures and limited oral health information provided at the child health clinic [31]. The complexity of parental struggles put young children at risk of poor oral health and contribute to structural inequalities [48]. We must tackle these structural barriers to help parents improve their children’s oral health [14].

Third, parents wish for help in raising their children backed up by more support in the wider social, community and professional environment. According to parent’s perspectives, there is no one simple solution to address the complex and interrelated problems of young children’s oral health problems. These findings align with the identified opportunities by Duijster and colleagues (2015) and van Nes and others (2018). Both studies show that parents consider child health clinics, dental practices, schools, and social welfare organisations important in promoting children’s oral health [31, 42]. Isong and co-researchers (2014) proposed various multilevel strategies, including an interdisciplinary partnership providing a stronger family support network [41]. Burgette and others (2023) stress the importance of communication between mothers and grandparents assisted by dental care professionals [46]. Groottens-Wiegers et al. (2020) demonstrated that parents perceived conversations with other peers as helpful because they found recognition of their experiences and parenting issues [49]. To improve children’s oral health, we must collaborate with families, their social surroundings, the community and professionals within and outside the dental sector.

### Strength and limitations

A key strength of our study is the active and participatory approach. We aimed to be part of the community by joining different activities organised by local societal organisations. At the start of the project, we invested in building warm relationships with parents rather than jumping straight to our research goals. By using this approach, we were able to talk to a diverse group of parents about a topic that may not be of immediate interest to them. Another strength is the use of multiple data collection methods. We used data triangulation to deepen our understanding of the data and increase the validity and credibility of our findings. However, our data collection methods are limited in gaining in-depth information from parents: it was difficult to estimate to what extent parents lived in vulnerable circumstances. The researcher’s position might have influenced our research findings. Parents may have given socially desirable answers when discussing topics related to poverty. We only received indirect information on poverty, such as expensive groceries or living in small and old houses. Also, our data was not suitable for conducting an in-depth analysis of how family composition, household functioning, occupation, cultural background, social network or living environment might influence parents’ perspectives on their children’s oral health. Despite many attempts to include fathers in our study, their perspectives are underrepresented. Since fathers are becoming more involved in parenting, studying fathers’ perspectives is important in promoting children’s oral health [50].

This qualitative study has important implications for children’s oral health promotion. First, we emphasise the importance of listening to parents’ situational needs and translating their proposed opportunities into practice [51]. Owen and co-workers (2022) showed that co-designing an oral health intervention based on families’ opinions increased supervised tooth brushing in children aged 9–12 months [52]. Translating parental knowledge into practice is important, given the increasing tendency for parents to refuse to participate in research. Parents may feel their participation is useless as their input disappears into the academic literature [53]. To do right to the parental perspectives of our study, a system approach seems most promising for child oral health promotion [43, 54]. We should stop placing the responsibility solely on the parents and start working towards the idea that children’s oral health is a shared responsibility [48]. The social context should consider their actions towards young children and how they can support parents in promoting oral health. Professionals should take responsibility and aim to provide parents with more coherent oral health advice. A more child-friendly approach is recommended for general dentists. The Group Care (GC) model offered by child health clinics) model is a promising approach that fits parents’ wish for more help in parenting and managing their hectic family life [55]. Such a GC model helps strengthen families in parenting through conversations with peers from their social surroundings while backed by a professional leading the group [56]. Also, creating healthier living environments is crucial to making a healthy diet an easy choice [57]. Finally, we believe that our findings will contribute to the WHO’s Global Oral Health Action Plan: “*Addressing oral health inequalities is a matter of social justice, ethical public health policy and professional practice*” (WHO, 2022, p.22), by using parents’ experiential knowledge as a starting point in developing new interventions [1].

Our results offer insight into the perspectives of a seldom-heard group and directions for future research lines. The intersection between social practices and oral health inequalities deserves deeper exploration [32]. Social practices are habits related to daily practices (i.e., sleeping, drinking, eating, moving, family, social and professional interactions) and are constituted by structural, social and material factors [58]. Identifying the social practices of oral health, including the relationships between these practices and other daily practices, is useful for a deeper understanding of why social practices undermining oral health are reproduced [32]. This knowledge is crucial for developing interventions that better address parenting and oral health needs. Designing a focused ethnographic study can deepen our understanding of the social practices and their inter-relationships impacting young children’s oral health [59]. For future research, we recommend using an intersectionality approach as a methodological analysis framework to understand better the various intersections underlying oral health inequalities [28].

To conclude, parents state that the problems underlying poor oral health in children (≤ 4 years) are complex and interrelated across family, societal, community and professional levels. They state that simply giving less candy and stimulating tooth brushing is not the solution to this complex problem. Using the experiential knowledge of parents might be the first step in acting upon the endeavour to address oral health inequality among young children [33, 53]. Based on the insights of this study, we will co-design interventions together with parents and professionals that fit the family’s perspectives and situational needs. Considering family’s understanding of oral health problems is a starting point in co-designing interventions to address persistent oral health inequalities.

### Electronic supplementary material

Below is the link to the electronic supplementary material.

Supplementary Material 1

Supplementary Material 2

## Data Availability

The anonymised dataset (transcripts and coding framework), semi-structured interview guide and tailored work plan for participant observations and focus group discussion are available from the corresponding author upon reasonable request.
