# Supplementary material for: Understanding parental perspectives on young children’s oral health (≤ 4 years) growing up in a disadvantaged neighbourhood of Amsterdam, the Netherlands: an exploratory study
Source: BMC Public Health. 2024 Feb 27;24:627. doi: 10.1186/s12889-024-18073-0 (PMC10900557; doi:10.1186/s12889-024-18073-0)
Supplement: Supplementary file 1 — Supplementary Material 1 [file 12889_2024_18073_MOESM1_ESM.docx]

**Addtional file 1.** Overview of participants.

| **Data collection method** | **Respondent** | **Mother(M) / Father(F)** | **Migration background** | **Single-/two parent family** | **# children** | **Occupation** | **Inclusion via** |
| --- | --- | --- | --- | --- | --- | --- | --- |
| Interviews | R1 | M | Brazilian | Two parents | 1 | Employed | Preschool |
|  | R2 | M | Moroccan | Single parent | 1 | Unemployed | Mosque |
|  | R3 | M | Moroccan | Married, two parents | 3 | Employed, shop assistant | Preschool |
|  | R4 | M | Dutch | Two parents | 1 | Employed, own company | Library |
|  | R5 | F | Turkish | Two parents | 3 | Employed, own company | Personal network |
|  | R6 | M | Moroccan | Two parents | 2 | Employed, pedagogical employee | Mosque |
|  | R7 | M | Unknown | Two parents | 3 | Unknown | Preschool |
|  | R8 | F | Dutch | Two parents | 1 | Employed, pedagogical employee | Library |
|  | R9 | M | Moroccan | Two parents | 3 | Employed | Personal network |
|  | R10 | M | Moroccan | Married, two parents | 3 | Parttime employed | Personal network |
|  | R11 | M | Moroccan | Married, two parents | 2 | Unemployed | Personal network |
|  | R12 | F | Turkish | Married, two parents | 1 | Employed | Personal network |
|  | R13 | M | Moroccan | Married, two parents | 2 | Unemployed | Personal network |
|  | R14 | M | Surinam/Dutch | Married, two parents | 7 | Employed, sport coach & health ambassador | Personal network |
|  | R15 | M | Moroccan | Married, two parents | 2 | Employed, youth worker | Mosque |
|  | R16 | M | Syrian | Two parents | 5 | Unemployed | Societal organisation 1 |
|  | R17 | M | Moroccan | Two parents | 2 | Unemployed | Personal network |
|  | R18 | M | Syrian | Two parents | 3 | Unemployed | Play group |
|  | R19 | M | Philippian | Two parents | 1 | Previously employed | Play group |
|  | R20 | M | Turkish | Two parents | 1 | Employed, banking employee | Personal network |
|  | R21 | M | Unknown | Two parents | 6 | Unemployed | Paediatric dental practice |
|  | R22 | M | Turkish | Two parents | 3 | Unemployed | Play group |
|  | R23 | M | Turkish | Two parents | 4 | Employed, own company | Paediatric dental practice |
|  | R24 | F | Moroccan | Two parents | 5 | Unknown | Societal organisation 2 |
|  | R25 | F | Moroccan | Two parents | 3 | Employed, electrician | Paediatric dental practice |
|  | R26 | M | Moroccan | Two parents | 3 | Unemployed | Paediatric dental practice |
|  | R27 | M | Turkish | Two parents | 2 | Unknown | Preschool |
|  | R28 | M | Turkish | Two parents | 3 | Unknown | Preschool |
|  | R29 | M | Turkish | Two parents | 1 | Unknown | Preschool |
|  | R30 | M | Unknown | Unknown | 2 | Unknown | Preschool |
|  | R31 | M | Unknown | Unknown | 3 | Unknown | Preschool |
|  | R32 | M | Unknown | Single parent | 1 | Employed | Preschool |
| Participant observations | R33 | M | Unknown | Two parents | 2 | Unknown | Play group |
|  | R34 | M | Unknown | Two parents | 3 | Previously employed in preschool | Play group |
|  | R35 | M | Unknown | Two parents | 2 | Unknown | Play group |
|  | R36 | M | Unknown | Two parents | 2 | Unknown | Play group |
|  | R37 | M | Unknown | Unknown | 4 | Employed, coordinator in societal organisation | Societal organisation 3 |
|  | R38 | M | Unknown | Two parents | 4 | Unknown | Societal organisation 3 |
|  | R39 | M | Unknown | Unknown | 2 | Unknown | Societal organisation 3 |
|  | R40 | M | Unknown | Two parents | 4 | Employed, mouth hygienist | Societal organisation 3 |
|  | R41 | M | Unknown | Unknown | 3 | Unknown | Play group |
|  | R42 | M | Italian | Unknown | 1 | Unknown | Play group |
|  | R43 | M | Turkish | Two parents | 1 | Unemployed | Play group |
|  | R44 | M | Moroccan | Two parents | 1 | Unemployed | Play group |
|  | R45 | M | Unknown | Two parents | 2 | Parttime employed | Play group |
|  | R46 | M | Moroccan | Two parents | 1 | Parttime employed | Play group |
|  | R47 | M | Eritrean | Single parent | 3 | Unknown | Play group |
| Focus group discussion | R48 | M | Unknown | Unknown | 3 | Unknown | Preschool |
|  | R49 | M | Unknown | Unknown | 3 | Employed, pedagogical employee | Preschool |
|  | R50 | M | Moroccan | Unknown | 2 | Unknown | Preschool |
|  | R51 | M | Servia | Unknown | 4 | Employed, dental assistant | Preschool |
|  | R52 | M | Unknown | Unknown | 2 | Unknown | Preschool |
